# Supplementary material for: Structural insights into selective and dual antagonism of EP2 and EP4 prostaglandin receptors
Source: EMBO J. 2025 Oct 29;44(23):7242–62. doi: 10.1038/s44318-025-00611-0 (PMC12669672; doi:10.1038/s44318-025-00611-0)
Supplement: Supplementary file 1 — Appendix [file 44318_2025_611_MOESM1_ESM.pdf]

Appendix

Structural Insights into Selective and Dual Antagonism of EP2 and EP4 Prostaglandin Receptors

Yanli Wu[1#]*, Heng Zhang[1#], Jiuyin Xu[2#], Kai Wu[1], Wen Hu[1], Xinheng He[1], Gaoming Wang[3], Canrong Wu[4]*, H. Eric Xu[1,4,5]*

[1]State Key Laboratory of Drug Research, Shanghai Institute of Materia Medica, Chinese Academy of Sciences, Shanghai 201203, China.

[2]School of Life Science and Technology, ShanghaiTech University, 201210 Shanghai, China.

[3]Department of Biliary-Pancreatic Surgery, Renji Hospital, Shanghai Jiao Tong University School of Medicine, Shanghai 200025, China.

[4]Research Center for Medicinal Structural Biology, National Research Center for Translational Medicine at Shanghai, State Key Laboratory of Medical Genomics, Ruijin Hospital, Shanghai Jiao Tong University School of Medicine, Shanghai 200025, China

[5]University of Chinese Academy of Sciences, Beijing 100049, China.

[#]These authors contributed equally: Yanli Wu, Heng Zhang, Jiuyin Xu.

*Corresponding authors: H. Eric Xu (eric.xu@simm.ac.cn), Canrong Wu (wcr13215@rjh.com.cn), Yanli Wu (wuyanli@simm.ac.cn)

Table of Contents:

Appendix Figure S1	Page 2
Appendix Figure S2	Page 3
Appendix Figure S3	Page 4
Appendix Figure S4	Page 5
Appendix Figure S5	Page 6
Appendix Figure S6	Page 7
Appendix Figure S7	Page 8
Appendix Table S1	Page 9
Appendix Table S2	Page 10
Appendix Table S3	Page 11
Appendix Table S4	Page 12
Appendix Table S5	Page 13

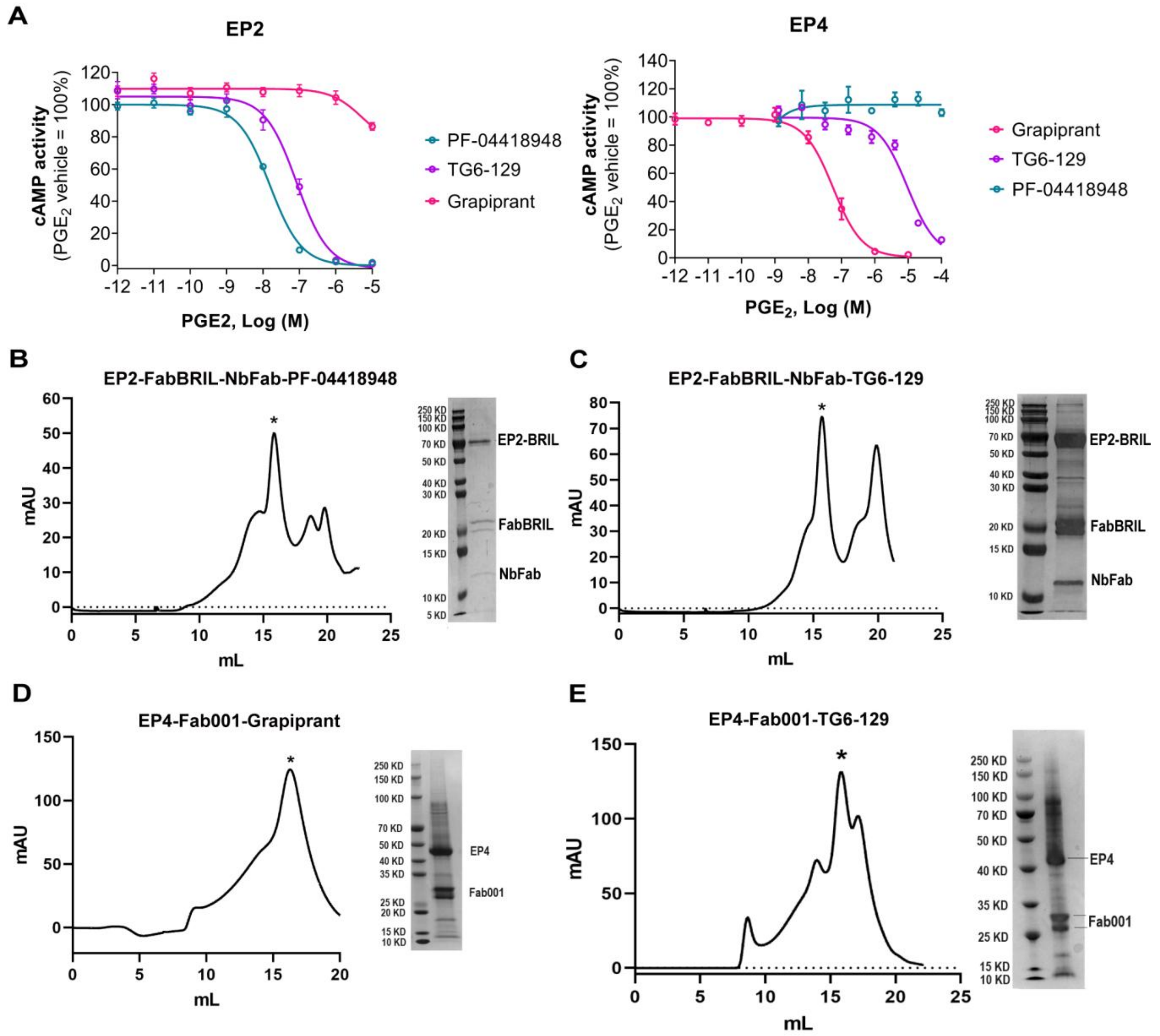

Appendix Figure S1 Ligands selectivity and purification of EP2-FabBRIL-NbFab-antagonist complexes.

A The antagonistic activity of the three different antagonists on EP2 (left) and EP4 (right) in PGE_2-induced cAMP assay. Data are presented as means ± S.E.M. of 3 independent experiments with 3 technical replicates respectively.

B-E Diagram of size-exclusion chromatography (left) and SDS-PAGE analysis (right) of EP2-FabBRIL-NbFab-PF-04418948 (B), EP2-FabBRIL-NbFab-TG6-129 (C), EP4-Fab001-grapiprant (D) and EP4-Fab001-TG6-129 complex (E), respectively.

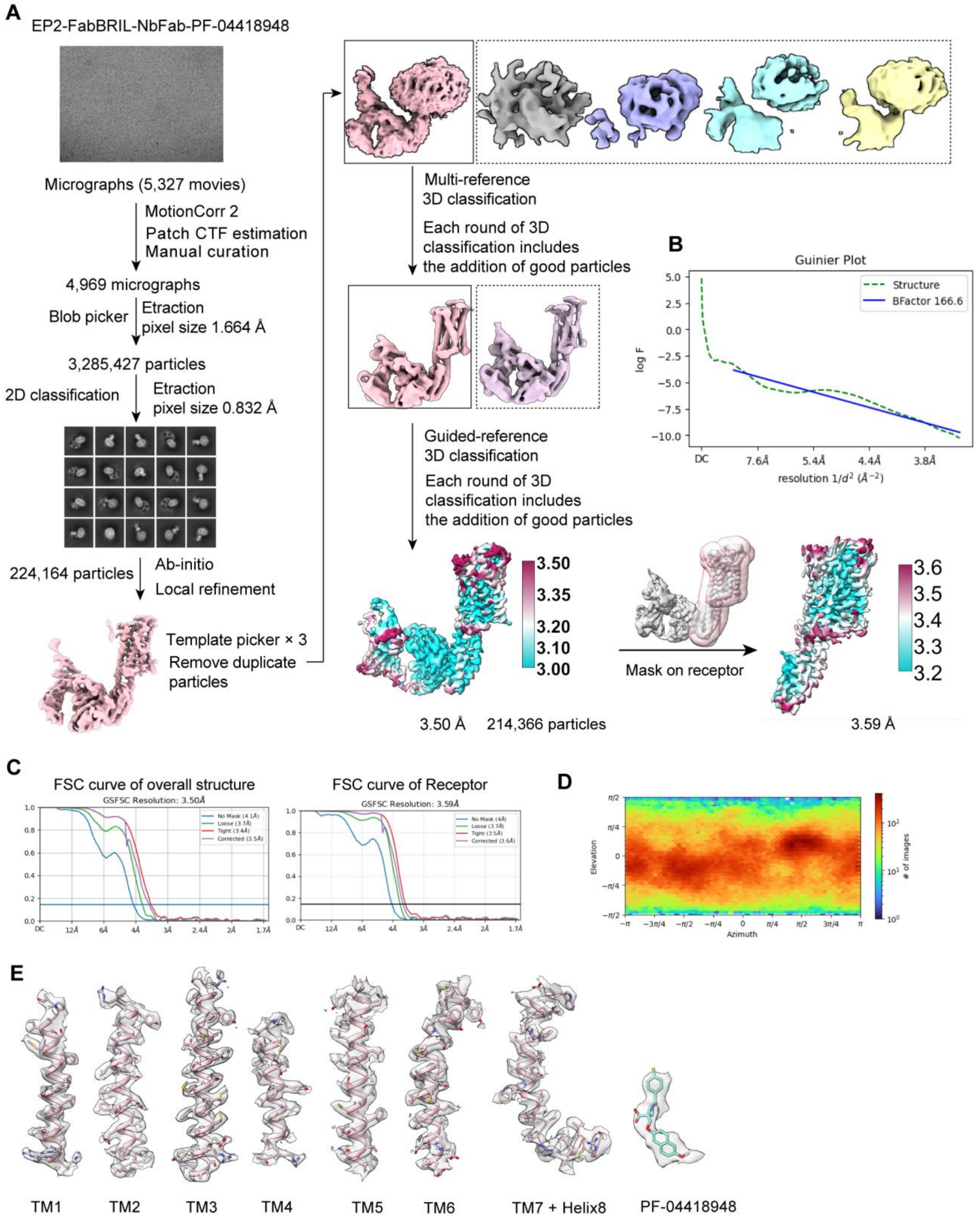

Appendix Figure S2 Cryo-EM data processing and representative cryo-EM density maps of EP2-FabBRIL-NbFab-PF-04418948 complex.

A Computational sorting of cryo-EM particle images.

B,C The sharping B factor and "Gold-standard" FSC curve for cryo-EM maps.

D The Euler angle distribution of final reconstructed local refinement map.

E Cryo-EM density maps of the TMs, helix8 and PF-04418948.

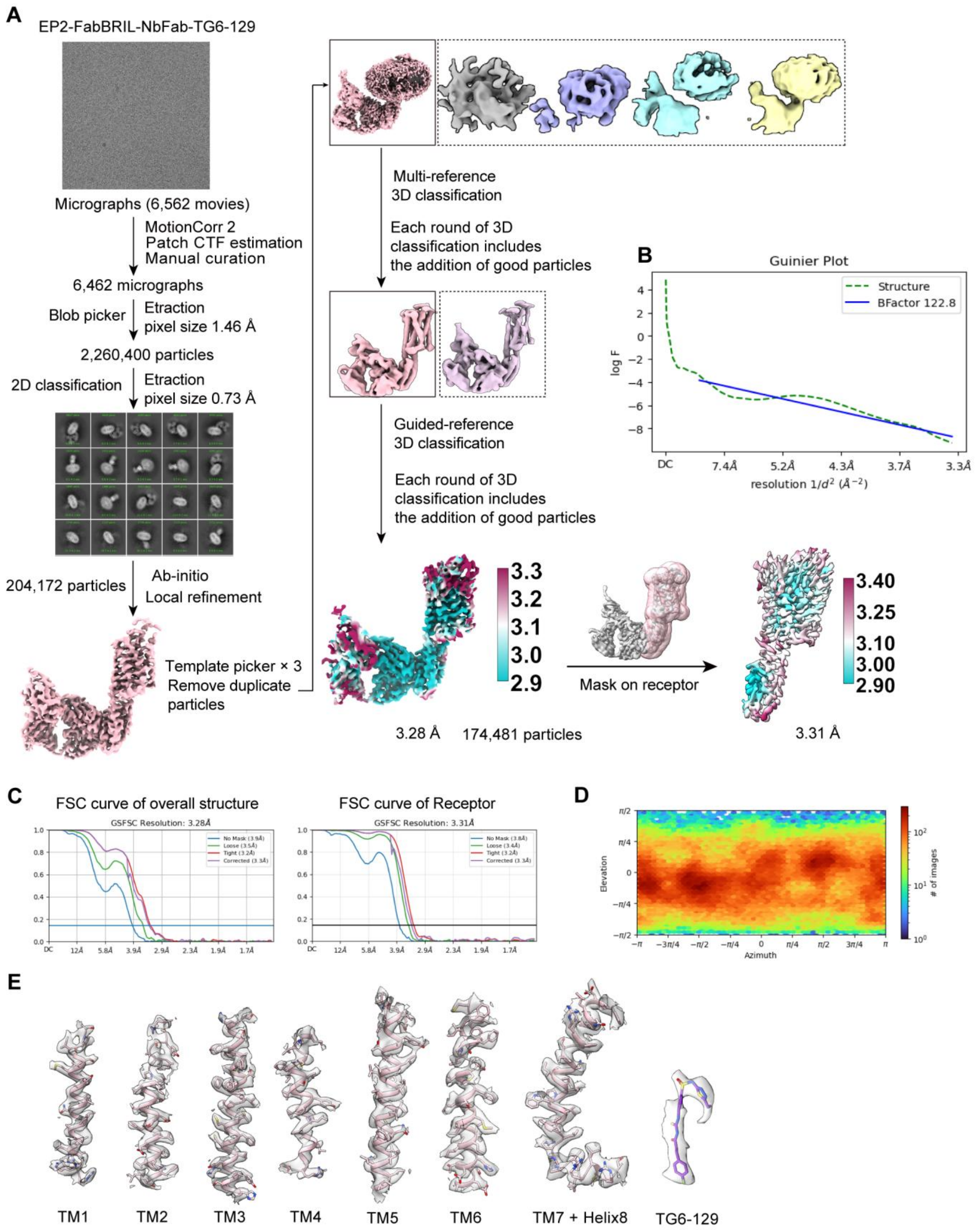

Appendix Figure S3 Cryo-EM data processing and representative cryo-EM density maps of EP2-FabBRIL-NbFab-TG6-129 complex.

A Computational sorting of cryo-EM particle images.

B,C The sharping B factor and "Gold-standard" FSC curve for cryo-EM maps.

D The Euler angle distribution of final reconstructed local refinement map.

E Cryo-EM density maps of the TMs, helix8 and TG6-129.

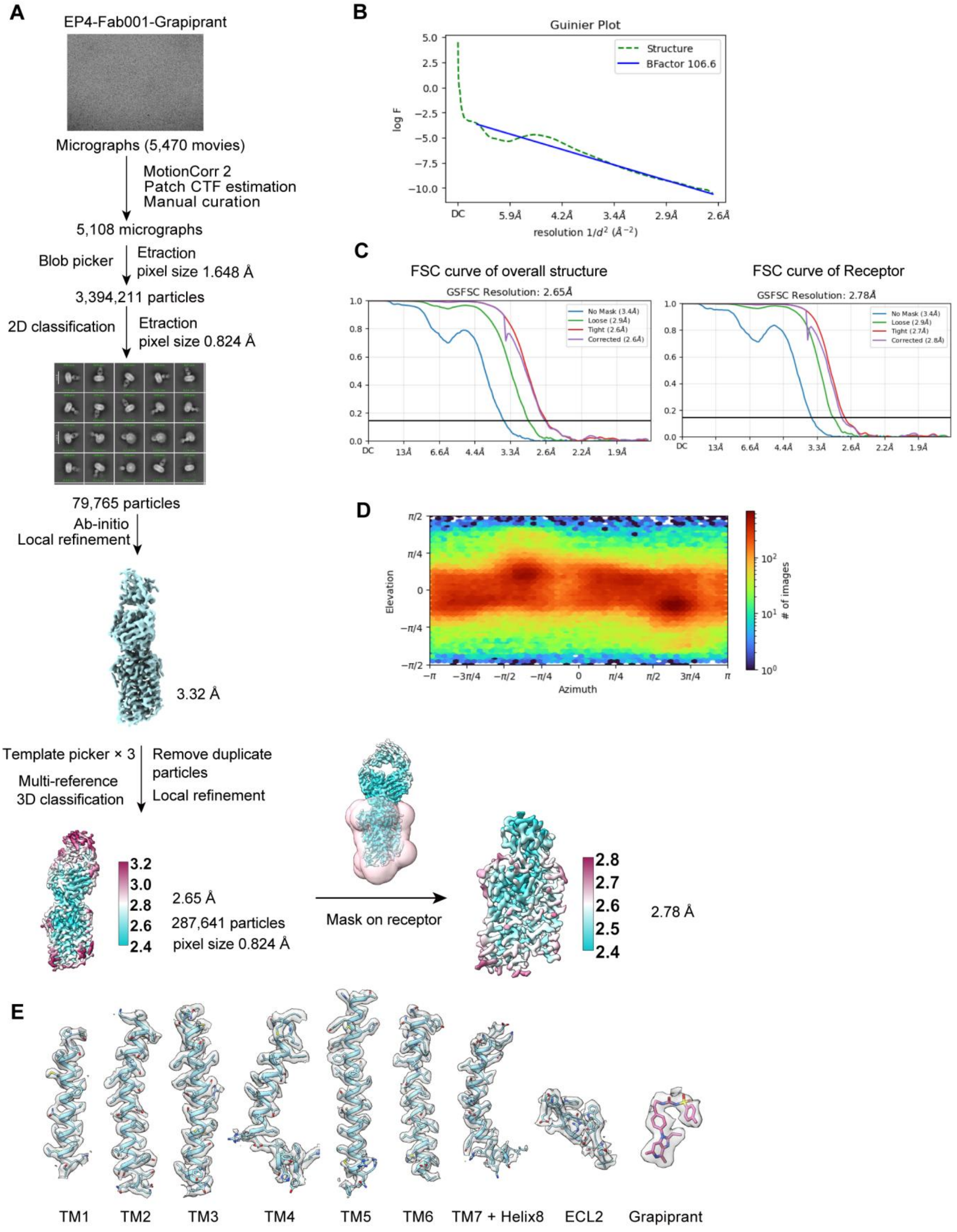

Appendix Figure S4 Cryo-EM data processing and representative cryo-EM density maps of EP4-Fab001-grapiprant complex.

A Computational sorting of cryo-EM particle images.

B,C The sharping B factor and "Gold-standard" FSC curve for cryo-EM maps.

D The Euler angle distribution of final reconstructed local refinement map.

E Cryo-EM density maps of the TMs, helix8, ECL2 and grapiprant.

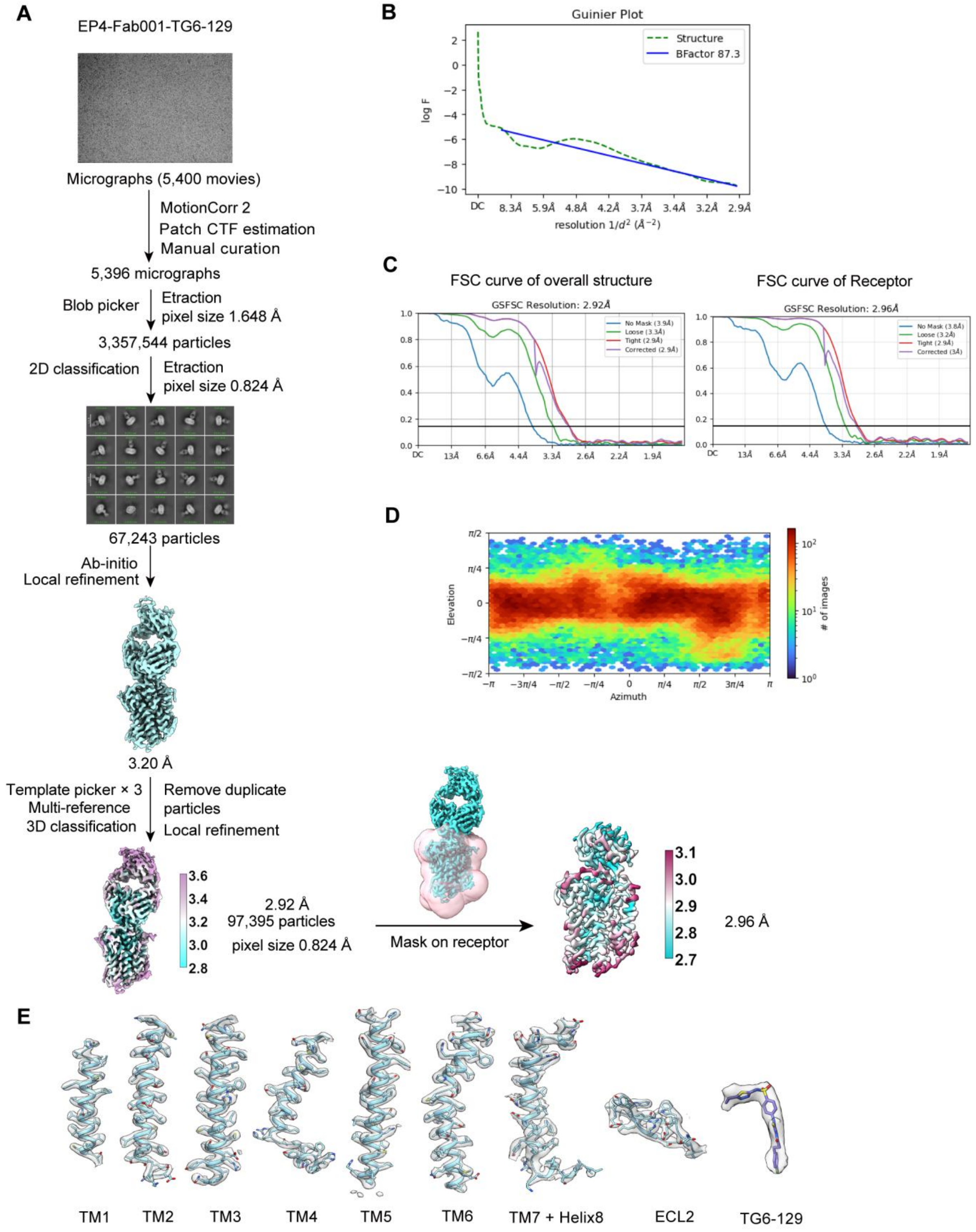

Appendix Figure S5 Cryo-EM data processing and representative cryo-EM density maps of EP4-Fab001-TG6-129 complex.

A Computational sorting of cryo-EM particle images.

B,C The sharping B factor and “Gold-standard” FSC curve for cryo-EM maps.

D The Euler angle distribution of final reconstructed local refinement map.

E Cryo-EM density maps of the TMs, helix8, ECL2 and TG6-129.

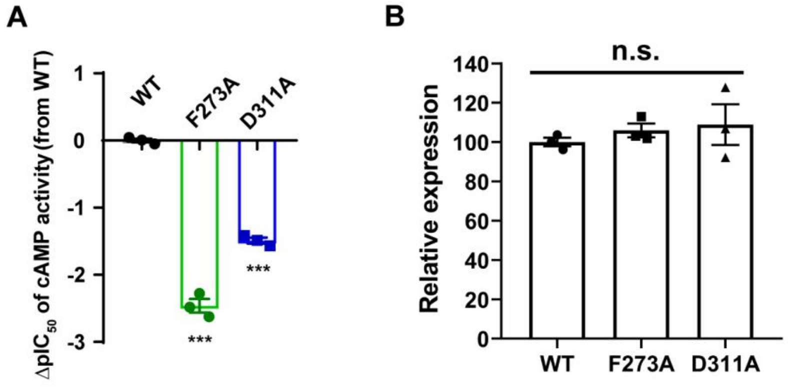

Appendix Figure S6 Additional data for activation of EP2.

A cAMP responses of key mutants in PGE_2 bound EP2. ΔpIC_{50} = pIC_{50} of PGE_2 to specific mutant - pIC_{50} of PGE_2 to WT. Data are presented as mean ± S.E.M. of 3 independent experiments with 3 technical replicates respectively. Significance was determined with a two-side unpaired t-test; ***$p<0.001$.

B Cell surface expression level of WT and mutant EP2 receptors. Data are presented as mean ± S.E.M. (n=3), significance was determined with two-side unpaired t test; $P>0.05$ was considered statistically no significant (n.s.).

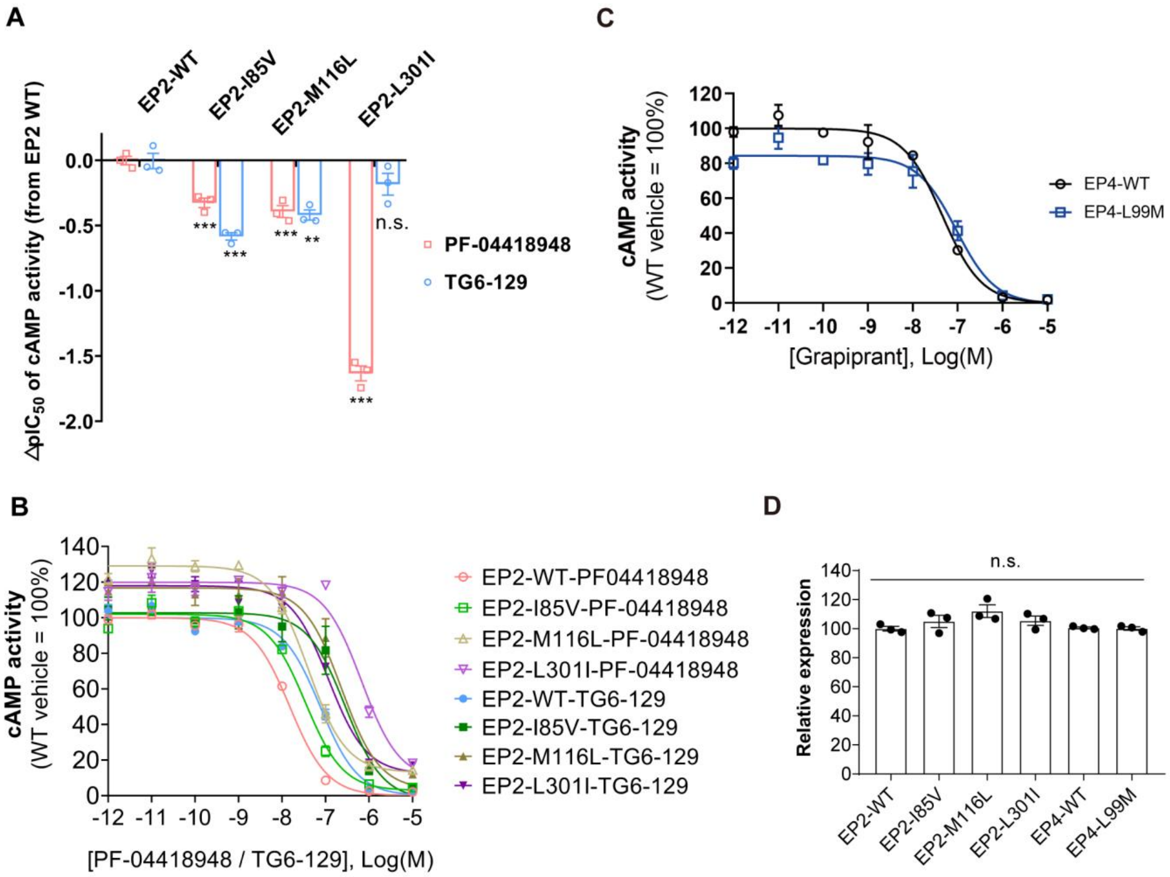

Appendix Figure S7 cAMP response in chimeric studies.

A,B ΔpIC_{50} (A) and cAMP response curve (B) for EP2 with the mutation of the semi-conserved residues. $\Delta pIC_{50} = pIC_{50}$ of antagonist to specific mutant - pIC_{50} to WT. Data are presented as mean ± S.E.M. of 3 independent experiments with 3 technical replicates respectively. Significance was determined with a two-side unpaired t-test; **$p<0.01$, ***$p<0.001$. $P>0.05$ was considered statistically no significant (n.s.).

C cAMP responses for EP4 with the mutation of the semi-conserved residue. Data are presented as mean ± S.E.M. of 3 independent experiments with 3 technical replicates respectively.

D Cell surface expression level of WT and mutant EP2, EP4 receptors. Data are presented as mean ± S.E.M. (n=3), significance was determined with two-side unpaired t test; $P>0.05$ was considered statistically no significant (n.s.).

Appendix Table S1

Table S1. Cryo-EM data collection, model refinement and validation statistics.

	EP2-PF04418948	EP2-TG6-129	EP4-Grapiprant	EP4-TG6-129
	PDB: 9JRO	PDB: 9JRT	PDB: 9JQZ	PDB: 9JQY
	EMDB: 61762	EMDB: 61763	EMDB: 61744	EMDB: 61743
**Data collection and processing**				
Magnification	105, 000	105, 000	105, 000	105, 000
Voltage (kV)	300	300	300	300
Electron exposure (e-/Å^2)	50	50	50	50
Defocus range (μm)	-1.2~-2.8	-1.2~-2.8	-1.2~-2.8	-1.2~-2.8
Pixel size (Å)	0.832	0.73	0.824	0.824
Symmetry imposed	C1	C1	C1	C1
Initial particle images (no.)	3,295,427	2,260,400	3,394,211	3,357,544
Final particle images (no.)	214,366	174,481	287,641	97,395
Overall Map resolution (Å)	3.50	3.28	2.65	2.92
Receptor Map resolution (Å)	3.59	3.31	2.78	2.96
FSC threshold	0.143			
Map sharping B factor (Å^2)	-166.6	-122.8	-106.6	-87.3
**Refinement**				
Initial mode used	From AlphaFold2	EP2-PF04418948	From AlphaFold2	EP4-Grapiprant
Model resolution (Å)	3.43	3.26	2.68	2.97
FSC threshold	0.143			
Model-Map CC (mask)	0.68	0.68	0.55	0.56
Model composition				
Non-hydrogen atoms	6955	7061	5398	5393
Protein residues	896	909	662	682
B factors (Å^2)				
Protein	61.60	61.71	36.39	36.40
Ligand	20.00	30.00	30.00	30.00
R.m.s.deviations				
Bond lengths	0.002	0.002	0.002	0.002
Bond angles	0.505	0.537	0.528	0.525
Validation				
MolProbity score	1.73	1.78	1.96	1.87
Clash score	6.53	6.64	6.34	5.51
Rotamer outliers (%)	3.27	3.41	4.31	4.81
Ramachandran plot				
Favored (%)	98.17	97.98	97.30	97.75
Allowed (%)	1.83	2.02	2.70	2.25
Disallowed (%)	0.00	0.00	0.00	0.00

Appendix Table S2

Table S2. PGE_2 induced cAMP signaling of EP2 assessed by GloSensor assay.

EC_{50} and E_{max} estimates represent the average and standard error of mean (S.E.M.) from n = 3 independent experiments with 3 technical replicates. E_{max} is defined as percentage of maximum response of wild type (WT). Statistical significance is indicated by p-value. (*** $p \leq 0.001$)

EP2	pEC_{50}	E_{max} (% WT)	ΔpEC_{50}	p-value of ΔpEC_{50}	Surface Expression (%)
WT	9.32 ± 0.12	100.0 ± 3.9	/	/	100.0 ± 2.2
F273A	6.86 ± 0.03	116.8 ± 2.9	-2.46 ± 0.10	0.0000***	105.9 ± 3.5
D311A	7.83 ± 0.08	92.4 ± 3.9	-1.49 ± 0.04	0.0000***	108.9 ± 10.4

Appendix Table S3

Table S3. The antagonism of PF-04418948 and TG6-129 on cAMP signaling of EP2 assessed by GloSensor assay.

EC_{50} and E_{max} estimates represent the average and standard error of mean (S.E.M.) from n = 3 independent experiments with 3 technical replicates. E_{max} is defined as percentage of maximum response of wild type (WT) respectively. Statistical significance is indicated by p-value. (* $p \leq 0.05$, ** $p \leq 0.01$, *** $p \leq 0.001$, n.s. $p > 0.05$)

Receptor	PGE_2	mutation	PF-04418948				TG6-129				
			pIC_{50}	E_{max} (% WT)	ΔpIC_{50}	p-value of ΔpIC_{50}	pIC_{50}	E_{max} (% WT)	ΔpIC_{50}	p-value of ΔpIC_{50}	Surface Expression (%)
EP2	1 nM	WT	8.10 ± 0.06	99.9 ± 1.4	/	/	7.09 ± 0.06	100.0 ± 1.4	/	/	100.0 ± 2.6
		E23A					7.69 ± 0.19	65.0 ± 3.2	0.56 ± 0.10	0.0063**	96.1 ± 2.1
		S28A					7.44 ± 0.19	94.1 ± 3.9	0.20 ± 0.06	0.0234*	106.8 ± 0.6
		T82A	7.56 ± 0.29	50.8 ± 2.6	-0.70 ± 0.11	0.00347**	7.13 ± 0.18	38.1 ± 1.6	-0.18 ± 0.03	0.0064**	108.8 ± 2.8
		S86A	8.36 ± 0.14	67.8 ± 2.1	-0.32 ± 0.06	0.00913**	6.47 ± 0.26	55.0 ± 2.9	-0.71 ± 0.06	0.0003***	97.0 ± 6.7
		M116A	7.08 ± 0.11	70.5 ± 1.4	-0.95 ± 0.13	0.00262**	6.29 ± 0.31	57.0 ± 3.1	-0.95 ± 0.02	0.0000***	101.8 ± 1.7
		W186A	6.64 ± 0.26	24.7 ± 0.4	-1.43 ± 0.14	0.00065***	5.70 ± 0.26	14.4 ± 0.6	-1.79 ± 0.34	0.0060**	94.8 ± 10.7
		S305A	6.98 ± 0.28	50.8 ± 2.4	-1.24 ± 0.14	0.00012***	7.54 ± 0.22	52.8 ± 2.6	0.47 ± 0.06	0.0034**	96.7 ±1.5
		S308A	7.09 ± 0.09	83.7 ± 1.5	-0.91 ± 0.09	0.00063***	6.68 ± 0.10	114.1 ± 2.8	-0.53 ± 0.02	0.0001***	105.6 ± 8.6
EP2	-	WT	8.03 ± 0.05	99.9 ± 1.3	/	/	7.06 ± 0.08	100.0 ± 1.8	/	/	
		E23A					7.50 ± 0.08	109.1 ± 2.1	0.36 ± 0.01	0.0023**	
		S28A					7.41 ± 0.08	274.1 ± 5.3	0.42 ± 0.02	0.0015**	
		T82A	7.67 ± 0.08	307.5 ± 6.0	-0.37 ± 0.10	0.03433*	6.66 ± 0.16	320.5 ± 10.8	-0.32 ± 0.08	0.0215*	
		S86A	7.72 ± 0.11	517.0 ± 13.9	-0.32 ± 0.03	0.00847**	6.22 ± 0.17	503.7 ± 9.9	-0.67 ± 0.07	0.0010**	
		M116A	6.51 ± 0.08	249.3 ± 4.3	-1.52 ± 0.12	0.00039***	6.31 ± 0.17	302.0 ± 6.7	-0.62 ± 0.01	0.0002***	
		W186A	5.99 ± 0.12	100.2 ± 2.0	-2.05 ± 0.09	0.00006***	5.44 ± 0.26	88.9 ± 2.6	-1.47 ± 0.16	0.0009***	
		S305A	7.01 ± 0.07	481.0 ± 7.4	-1.01 ± 0.02	0.00008***	7.30 ± 0.11	514.6 ± 12.3	0.38 ± 0.10	0.0296*	
		S308A	7.75 ± 0.09	84.3 ± 2.0	-0.28 ± 0.10	0.06985n.s.	7.23 ± 0.14	97.5 ± 3.3	0.25 ± 0.11	0.1455n.s.	

Appendix Table S4

Table S4. The antagonism of grapiprant and TG6-129 on cAMP signaling of EP4 assessed by GloSensor assay.

EC_{50} and E_{max} estimates represent the average and standard error of mean (S.E.M.) from n = 3 independent experiments with 3 technical replicates. E_{max} is defined as percentage of maximum response of wild type (WT) respectively. Statistical significance is indicated by p-value. (* $p \le 0.05$, ** $p \le 0.01$, *** $p \le 0.001$, n.s. $p > 0.05$)

Receptor	PGE_2	mutation	Grapiprant				TG6-129				
			pIC_{50}	E_{max} (% WT)	ΔpIC_{50}	p-value of ΔpIC_{50}	pIC_{50}	E_{max} (% WT)	ΔpIC_{50}	p-value of ΔpIC_{50}	Surface Expression (%)
EP4	0.5 nM	WT	7.45 ± 0.05	99.5 ± 1.2	/	/	5.02 ± 0.11	99.6 ± 2.3	/	/	100.0 ± 7.9
		V72A	6.96 ± 0.11	44.9 ± 1.3	-0.29 ± 0.06	0.0383*	4.74 ± 0.15	55.1 ± 1.4	-0.29 ± 0.09	0.0433*	105.6 ± 1.4
		S73A	8.25 ± 0.11	27.9 ± 0.9	0.99 ± 0.07	0.0001***					95.4 ± 10.0
		I317A					4.97 ± 0.12	56.2 ± 1.3	0.73 ± 0.07	0.4834n.s.	117.8 ± 2.7
		S319A	6.73 ± 0.16	37.9 ± 1.2	-0.64 ± 0.07	0.0013**	4.70 ± 0.26	45.8 ± 1.8	-0.30 ± 0.08	0.0322*	106.0 ± 9.1
		V320A	6.47 ± 0.27	47.1 ± 2.7	-0.78 ± 0.12	0.0013**	4.87 ± 0.14	71.6 ± 1.7	-0.11 ± 0.05	0.2566n.s.	99.7 ± 2.9
EP4	-	WT	8.37 ± 0.05	100.0 ± 1.6	/	/	5.30 ± 0.06	100.0 ± 1.4	/	/	
		V72A	7.73 ± 0.09	42.1 ± 1.0	-0.65 ± 0.08	0.0018**	4.73 ± 0.12	46.5 ± 1.0	-0.48 ± 0.02	0.0006***	
		S73A	8.66 ± 0.06	176.2 ± 3.0	0.22 ± 0.08	0.0635n.s.					
		I317A					5.45 ± 0.06	82.1 ± 1.3	0.26 ± 0.11	0.1223n.s.	
		S319A	7.78 ± 0.08	125.8 ± 2.7	-0.60 ± 0.09	0.0045**	4.70 ± 0.15	119.7 ± 3.6	-0.46 ± 0.02	0.0008***	
		V320A	7.95 ± 0.10	53.1 ± 1.5	-0.39 ± 0.12	0.0396*	5.17 ± 0.09	60.7 ± 1.2	-0.01 ± 0.04	0.5647n.s.	

Appendix Table S5

Table S5. The antagonism of ligands on EP2 and EP4 against PGE_2 induced cAMP signaling of EP2 assessed by GloSensor assay in chimeric studies.

pIC_{50} and E_{max} estimates represent the average and standard error of mean (S.E.M.) from n = 3 independent experiments with 3 technical replicates. E_{max} is defined as percentage of maximum response of wild type (WT). Statistical significance is indicated by p-value. (* $p \leq 0.05$, ** $p \leq 0.01$, *** $p \leq 0.001$, n.s. $p > 0.05$)

Ligand	Receptor	pIC_{50}	E_{max} (% WT)	ΔpIC_{50}	p-value of ΔpIC_{50}	Surface Expression (%)
PF-04418948	EP2-WT	7.82 ± 0.05	100.0 ± 1.3	/	/	100.0 ± 1.6
	EP2-I85V	7.47 ± 0.07	102.0 ± 1.8	-0.33 ± 0.04	0.0005***	105.0 ± 4.2
	EP2-M116L	7.40 ± 0.07	129.1 ± 1.9	-0.39 ± 0.04	0.0004***	112.0 ± 4.3
	EP2-L301I	6.17 ± 0.11	119.9 ± 2.2	-1.63 ± 0.06	0.0000***	105.4 ± 3.2
TG6-129	EP2-WT	7.12 ± 0.07	99.9 ± 1.7	/	/	
	EP2-I85V	6.55 ± 0.15	102.7 ± 3.1	-0.58 ± 0.03	0.0007***	
	EP2-M116L	6.63 ± 0.13	116.7 ± 3.0	-0.42 ± 0.04	0.0045**	
	EP2-L301I	6.93 ± 0.12	117.9 ± 2.9	-0.18 ± 0.08	0.1430n.s.	
Grapiprant	EP4-WT	7.34 ± 0.09	100.0 ± 2.2	/	/	100.6 ± 0.6
	EP4-L99M	7.03 ± 0.12	84.3 ± 2.5	-0.23 ± 0.07	0.0116	100.1 ± 1.2
