# Supplementary figures and images for: Structural insights into selective and dual antagonism of EP2 and EP4 prostaglandin receptors
Source: EMBO J. 2025 Oct 29;44(23):7242–62. doi: 10.1038/s44318-025-00611-0 (PMC12669672; doi:10.1038/s44318-025-00611-0)

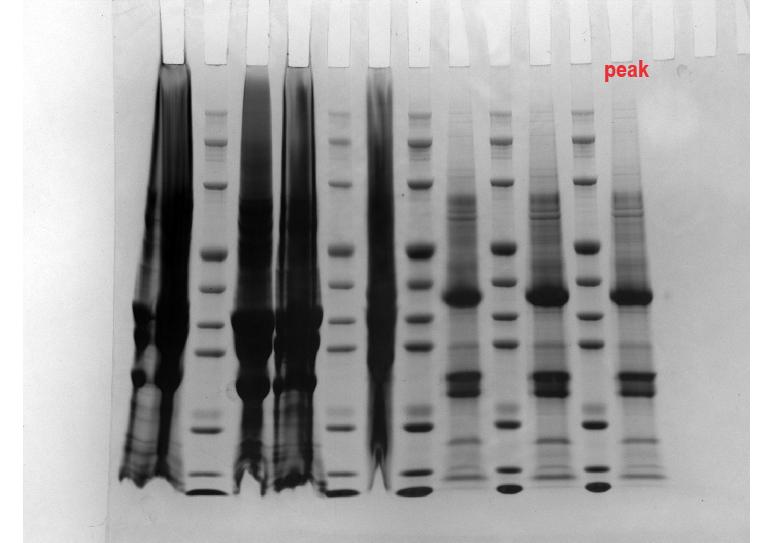

Supplement: Supplementary file 10 — Appendix Figure Source Data [file 44318_2025_611_MOESM10_ESM.zip › Appendix Figure S1/S1D/EP4-Fab001-Grapiprant SDS-PAGE.tif]

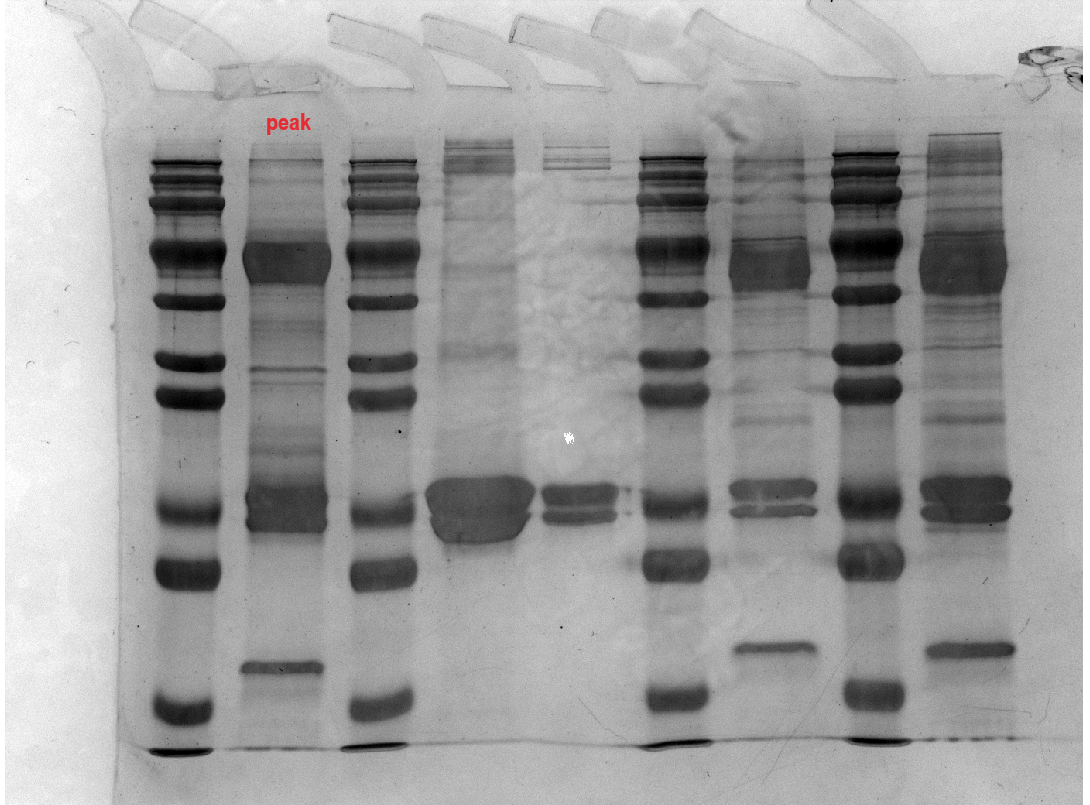

Supplement: Supplementary file 10 — Appendix Figure Source Data [file 44318_2025_611_MOESM10_ESM.zip › Appendix Figure S1/S1C/EP2-FabBRIL-NbFab-TG6-129 SDS-PAGE.tif]

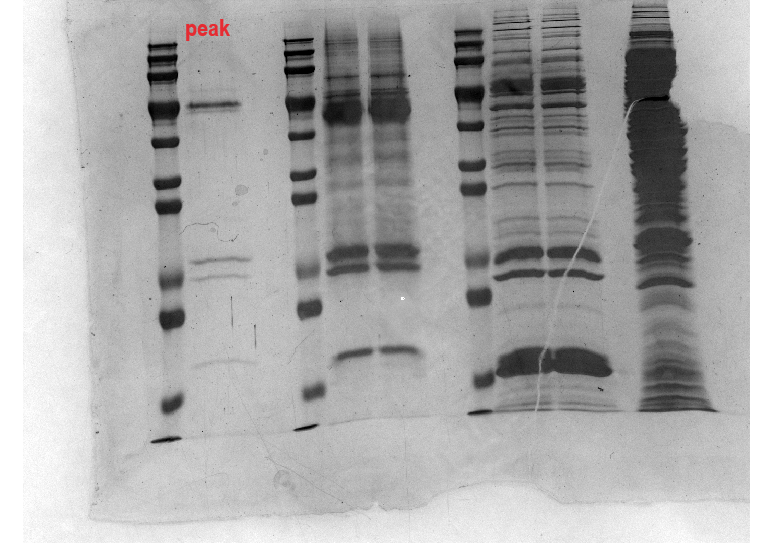

Supplement: Supplementary file 10 — Appendix Figure Source Data [file 44318_2025_611_MOESM10_ESM.zip › Appendix Figure S1/S1B/EP2-FabBRIL-NbFab-PF-04418948 SDS-PAGE.tif]

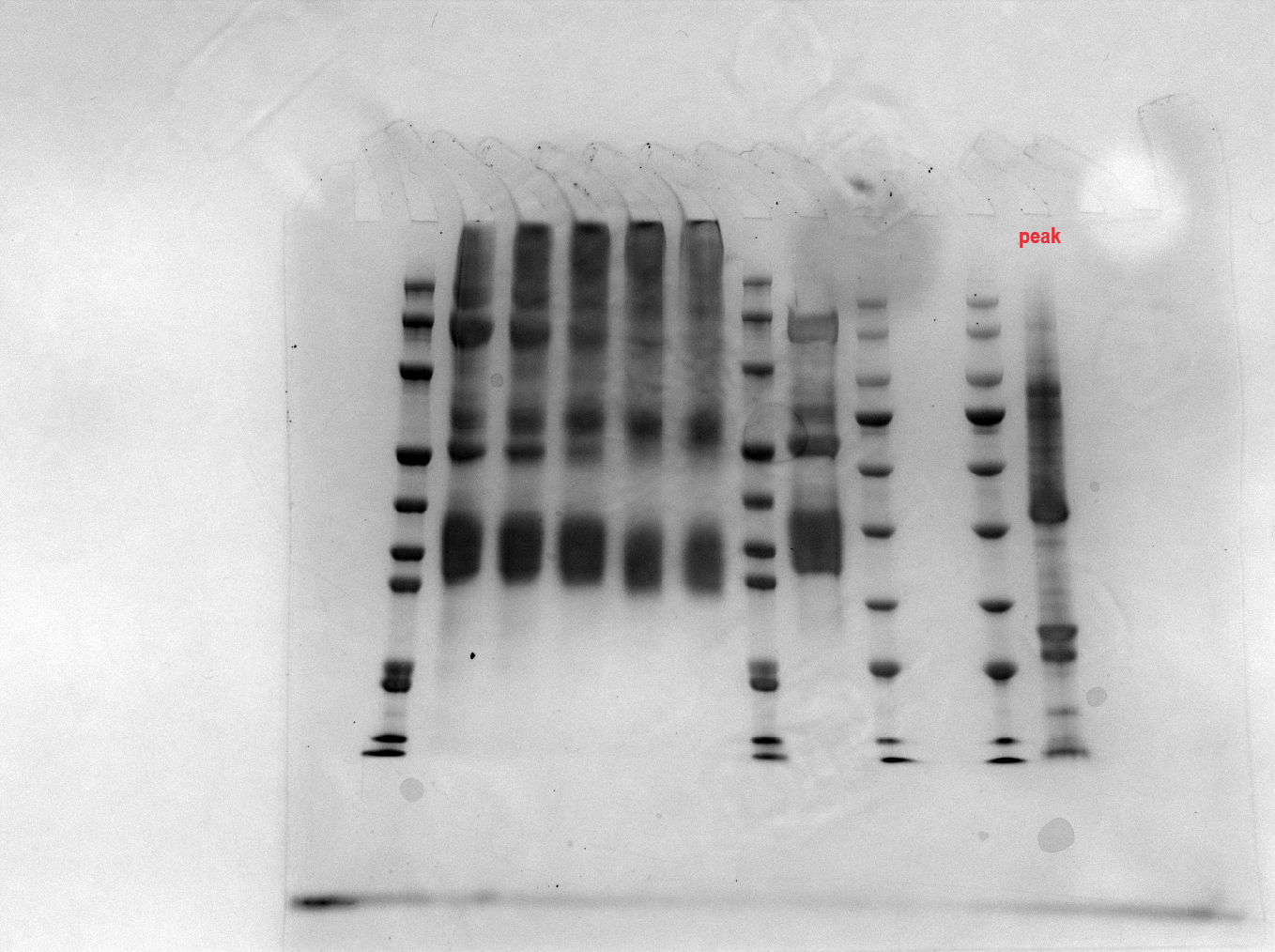

Supplement: Supplementary file 10 — Appendix Figure Source Data [file 44318_2025_611_MOESM10_ESM.zip › Appendix Figure S1/S1E/EP4-Fab001-TG6-129 SDS-PAGE.tif]
