# Supplementary material for: Structural insights into selective and dual antagonism of EP2 and EP4 prostaglandin receptors
Source: EMBO J. 2025 Oct 29;44(23):7242–62. doi: 10.1038/s44318-025-00611-0 (PMC12669672; doi:10.1038/s44318-025-00611-0)
Supplement: Supplementary file 11 — Expanded View Figures [file 44318_2025_611_MOESM11_ESM.pdf]

Expanded View Figures

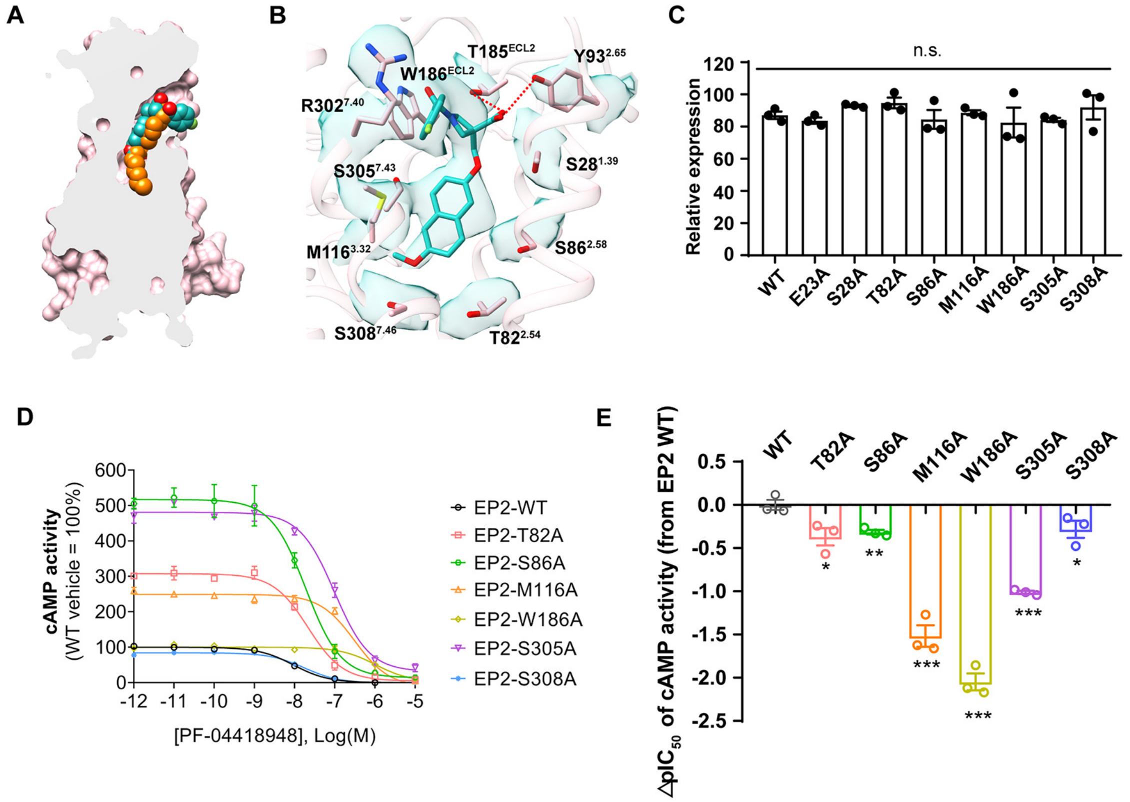

Figure EV1. Additional data for inhibition of EP2.

(**A**) Comparison of binding pocket of PF-04418948 (light green) and PGE_2 (orange) in inactive EP2 (light pink). (**B**) Cryo-EM density map of key residues in PF-04418948 binding pocket in EP2. (**C**) Cell surface expression level of WT and mutant EP2 receptors. Data are presented as mean ± S.E.M. ($n = 3$), significance was determined with two-side unpaired t test; $P > 0.05$ was considered statistically no significant (n.s.). (**D**, **E**) cAMP response (**D**) and ΔpIC_{50} (**E**) of PF-04418948 in constitutively active EP2 mutants. $\Delta pIC_{50} = pIC_{50}$ of PF-04418948 to specific mutant - pIC_{50} of PF-04418948 to WT. Data are presented as mean ± S.E.M. of 3 independent experiments with 3 technical replicates, respectively. Significance was determined with a two-sided unpaired t test. Compared with EP2 WT, the P values in panel E are 0.0343, 0.0085, 0.0004, 0.0001, 0.0001 and 0.0699 for EP2 T82A, S86A, M116A, W186A, S305A and S308A mutant, respectively. $^*P < 0.05$, $^{**}P < 0.01$, $^{***}P < 0.001$. Source data are available online for this figure.

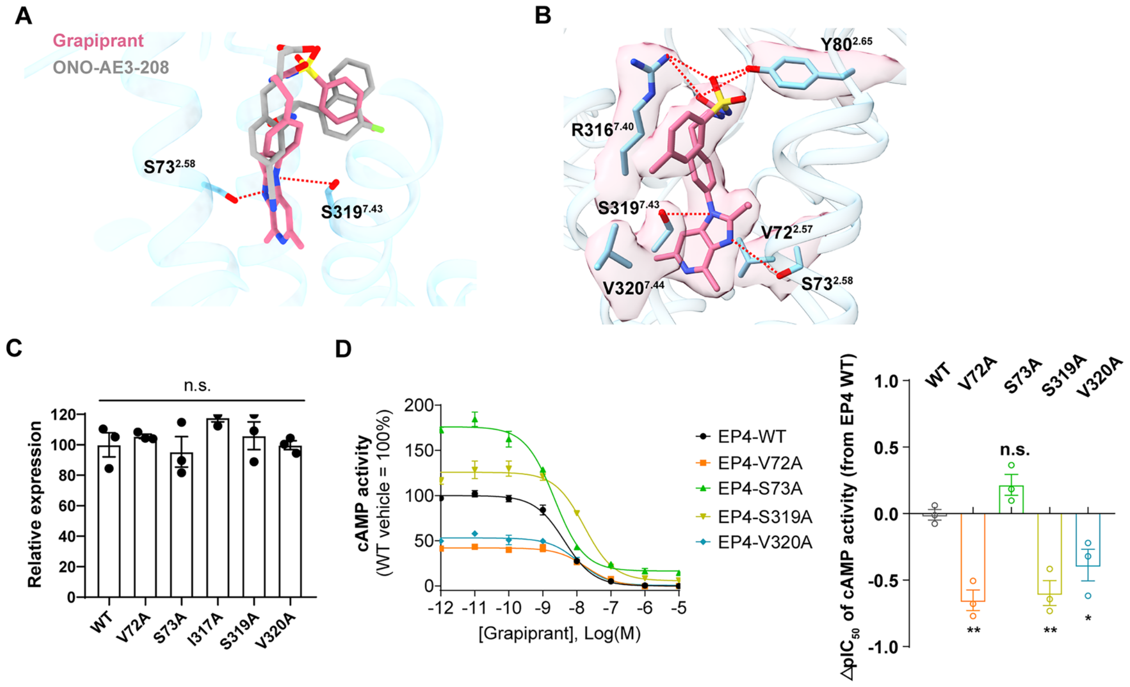

Figure EV2. Additional data for selective inhibition of EP4.

(**A**) Comparison of binding pocket of grapiprant and ONO-AE3-208 in inactive EP4 (light blue). (**B**) Cryo-EM density map of key residues in grapiprant binding pocket in EP4. (**C**) Cell surface expression level of WT and mutant EP4 receptors. Data are presented as mean ± S.E.M. ($n = 3$), significance was determined with two-sided unpaired *t* test; $P > 0.05$ was considered statistically no significant (n.s.). (**D**) cAMP response (left) and ΔpIC_{50} (right) of grapiprant in constitutively active EP4 mutants. $\Delta pIC_{50} = pIC_{50}$ of PF-04418948 to specific mutant - pIC_{50} of grapiprant to WT. Data are presented as mean ± S.E.M. of 3 independent experiments with 3 technical replicates, respectively. Significance was determined with a two-sided unpaired *t* test. Compared with EP4 WT, the *P* values in the right panel are 0.0018, 0.0635, 0.0045 and 0.0396 for EP4 V72A, S73A, S319A and V320A mutant, respectively. $^*P < 0.05$, $^{**}P < 0.01$, $^{***}P < 0.001$. Source data are available online for this figure.

 © The Author(s)

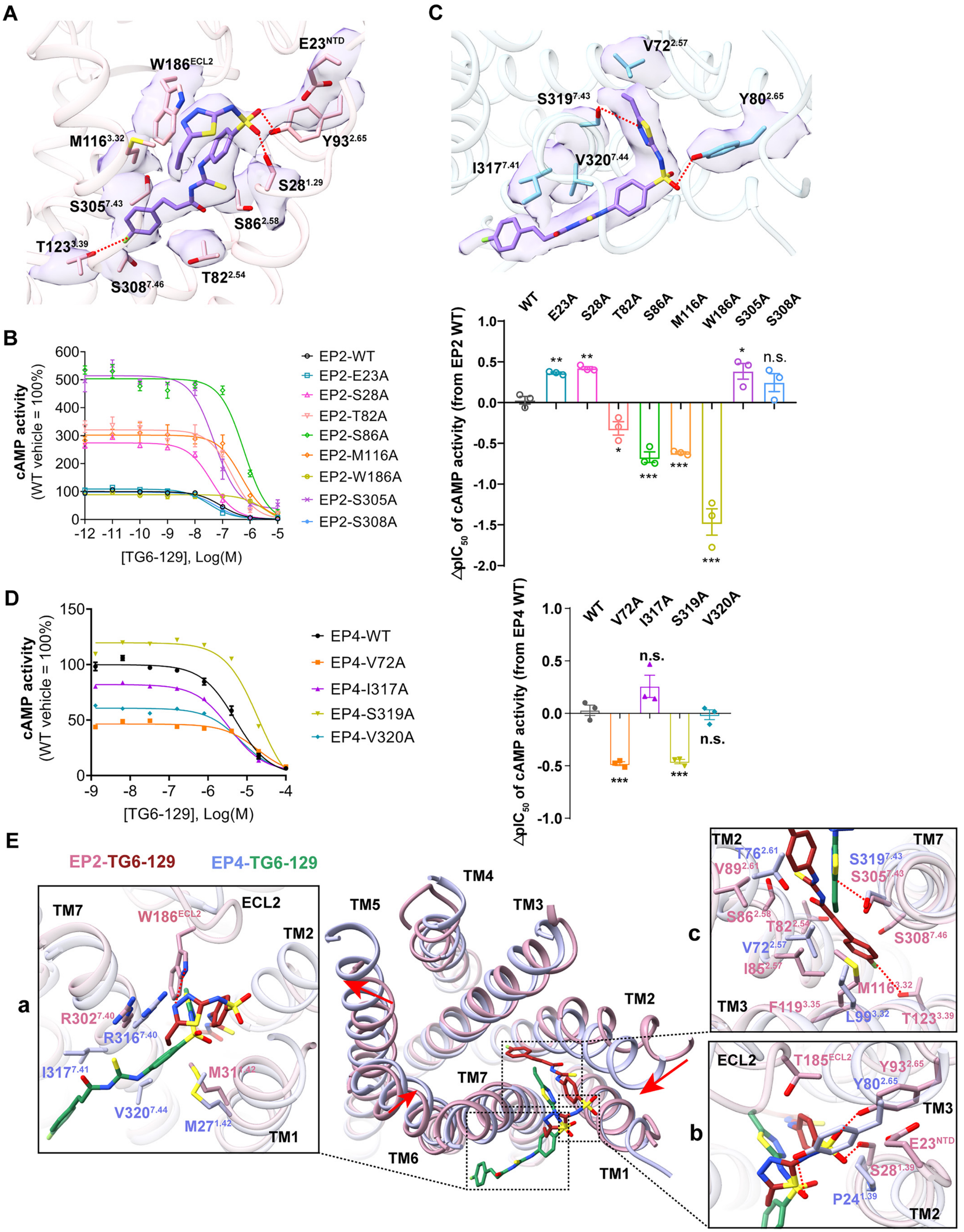
A
W186ECL2
E23NTD
M1163.32
Y932.65
S281.29
S3057.43
S862.58
T1233.39
S3087.46
T822.54
B
cAMP activity (WT vehicle = 100%)
EP2-WT
EP2-E23A
EP2-S28A
EP2-T82A
EP2-S86A
EP2-M116A
EP2-W186A
EP2-S305A
EP2-S308A
[TG6-129], Log(M)
C
V722.57
S3197.43
Y802.65
I3177.41
V3207.44
ΔpIC50 of cAMP activity (from EP2 WT)
WT E23A S28A T82A S86A M116A W186A S305A S308A
n.s.
D
cAMP activity (WT vehicle = 100%)
EP4-WT
EP4-V72A
EP4-I317A
EP4-S319A
EP4-V320A
[TG6-129], Log(M)
ΔpIC50 of cAMP activity (from EP4 WT)
WT V72A I317A S319A V320A
n.s.
E
EP2-TG6-129
EP4-TG6-129
a
b
c
TM1
TM2
TM3
TM4
TM5
TM6
TM7
ECL2
W186ECL2
R3027.40
R3167.40
I3177.41
V3207.44
M271.42
T762.61
V892.61
S3197.43
S3057.43
S862.58
T822.54
V722.57
I852.57
S3087.46
M1163.32
F1193.35
L993.32
T1233.39
T185ECL2
Y932.65
Y802.65
E23NTD
S281.39
P241.39

Figure EV3. Additional data for dual inhibition of EP2 and EP4.

(**A**) Cryo-EM density map of key residues in TG6-129 (purple) binding pocket in EP2 (light pink). (**B**) cAMP response (left) and ΔpIC_{50} (right) of TG6-129 in constitutively active EP2 mutants. $\Delta pIC_{50} = pIC_{50}$ of TG6-129 to specific mutant - pIC_{50} of TG6-129 to WT. Compared with EP2 WT, the *P* values in the right panel are 0.0023, 0.0015, 0.0215, 0.0010, 0.0002, 0.0009, 0.0296 and 0.1455 for EP2 E23A, S28A, T82A, S86A, M116A, W186A, S305A and S308A mutant, respectively. (**C**) Cryo-EM density map of key residues in TG6-129 binding pocket in EP4 (light blue). (**D**) cAMP response (left) and ΔpIC_{50} (right) of TG6-129 in constitutively active EP4 mutants. Compared with EP4 WT, the *P* values in the right panel are 0.0006, 0.1223, 0.0008 and 0.5647 for EP4 V72A, I317A, S319A and V320A mutant, respectively. (**E**) Structure comparison of TG6-129 (brown) bound EP2 and TG6-129 (green) bound EP4. The conformational changes of top view (middle) are depicted by red arrows, and the comparison of key residues in the outside (a), top (b) and inside (c) of the binding pocket are shown in a zoomed-in view. Data information: Data are presented as mean ± S.E.M. of 3 independent experiments with 3 technical replicates respectively. Significance was determined with a two-sided unpaired *t* test; $^{*}P \le 0.05$, $^{**}P \le 0.01$, $^{***}P \le 0.001$, n.s. $P > 0.05$. Source data are available online for this figure.

© The Author(s)

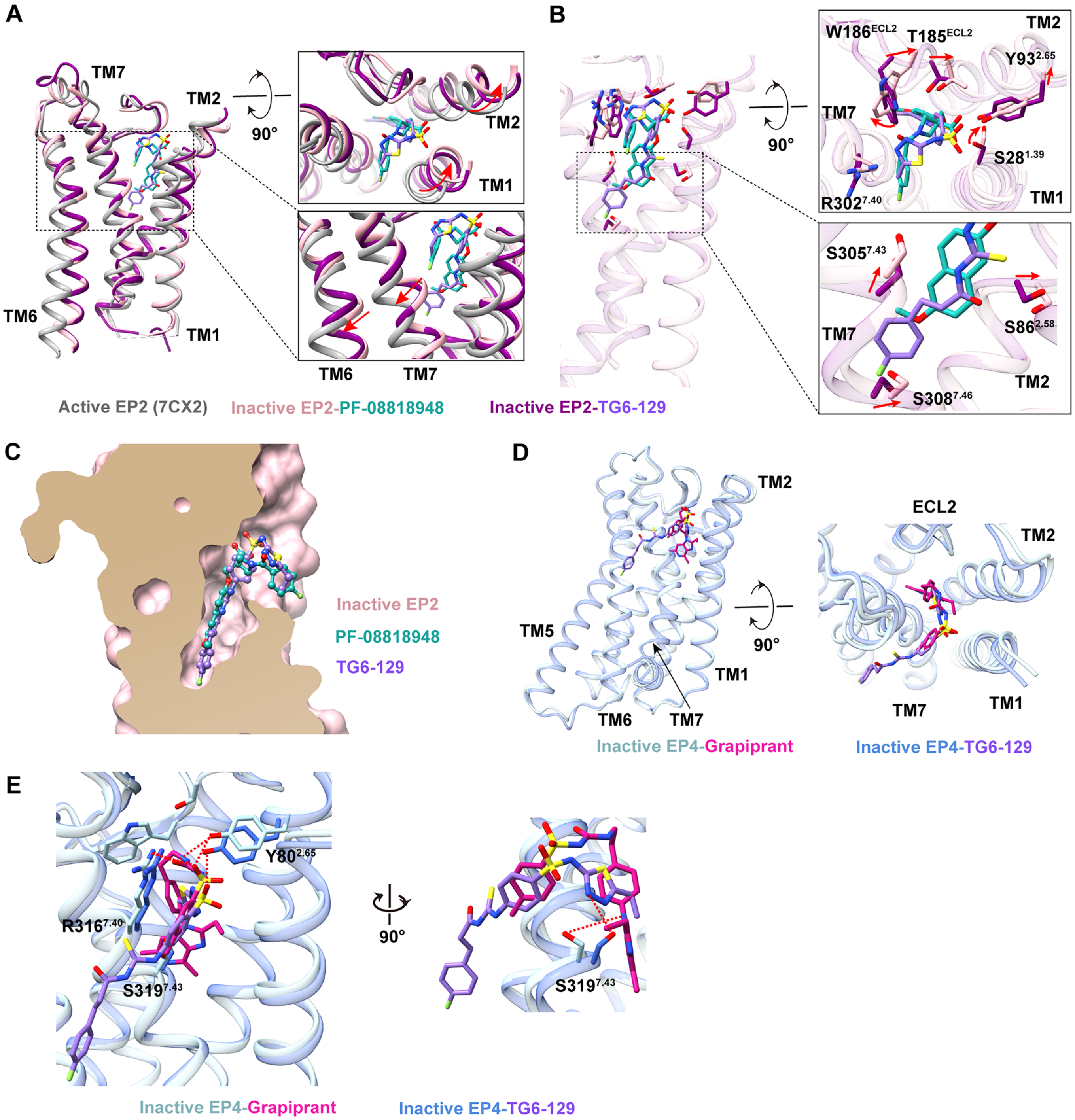

Figure EV4. Additional data for rational design of selective and dual EP2 and EP4 antagonists.

(**A**) Structure comparison of active EP2, inactive PF-04418948-EP2 and inactive TG6-129-EP2 complexes. The conformational changes of top view (upper right) and in the TM6, TM7 (bottom right) are depicted by red arrows in a zoomed-in view. (**B**) Comparison of the key residues in EP2 interacting with PF-04418948 and TG6-129. The residue position and conformational changes of in the top of the pocket (upper right) and in the TM6, TM7 (bottom right) are depicted by red arrows in a zoomed-in view. (**C**) Comparison of binding pocket of PF-04418948 and TG6-129 in inactive EP2. (**D**) Side view (left) and top view (right) of structure comparison between inactive grapiprant-EP4 and inactive TG6-129-EP4 structures. (**E**) Comparison of the key residues in EP2 interacting with PF-04418948 and TG6-129 in the front (left) and side (right) view.
